# Resilience of marine invertebrate communities during the early Cenozoic hyperthermals

**DOI:** 10.1038/s41598-020-58986-5

**Published:** 2020-02-07

**Authors:** William J. Foster, Christopher L. Garvie, Anna M. Weiss, A. D. Muscente, Martin Aberhan, John W. Counts, Rowan C. Martindale

**Affiliations:** 1 Museum für Naturkunde, Leibniz Institute for Evolution and Biodiversity, Invalidenstraße 43, Berlin, 10115 Germany; 2 University of Potsdam, Institute for Geosciences, Karl-Liebknecht Straße 24-25, Potsdam-Golm, 14476 Germany; 3 University College Dublin, School of Earth Sciences, Belfield, Dublin 4 Ireland; 4 Non-Vertebrate Paleontology Laboratory, Texas Natural Science Center, The University of Texas at Austin, 10100 Burnet Road, Austin, Texas 78758 USA; 5 The University of Texas at Austin, Department of Geological Sciences, 2275 Speedway, Austin, Texas 78712 USA; 6 Cornell College, Department of Geology, Mount Vernon, Iowa 600 First Street SW, 52314 USA

**Keywords:** Climate-change impacts, Climate-change ecology

## Abstract

The hyperthermal events of the Cenozoic, including the Paleocene-Eocene Thermal Maximum, provide an opportunity to investigate the potential effects of climate warming on marine ecosystems. Here, we examine the shallow benthic marine communities preserved in the late Cretaceous to Eocene strata on the Gulf Coastal Plain (United States). In stark contrast to the ecological shifts following the end-Cretaceous mass extinction, our data show that the early Cenozoic hyperthermals did not have a long-term impact on the generic diversity nor composition of the Gulf Coastal Plain molluscan communities. We propose that these communities were resilient to climate change because molluscs are better adapted to high temperatures than other taxa, as demonstrated by their physiology and evolutionary history. In terms of resilience, these communities differ from other shallow-water carbonate ecosystems, such as reef communities, which record significant changes during the early Cenozoic hyperthermals. These data highlight the strikingly different responses of community types, i.e., the almost imperceptible response of molluscs versus the marked turnover of foraminifera and reef faunas. The impact on molluscan communities may have been low because detrimental conditions did not devastate the entire Gulf Coastal Plain, allowing molluscs to rapidly recolonise vacated areas once harsh environmental conditions ameliorated.

## Introduction

Human activities are drastically changing conditions in coastal marine ecosystems by polluting, destroying habitats, overexploiting resources, enabling invasive species, and driving climate warming. Increased greenhouse gas emissions associated with these activities harm marine communities by expanding hypoxic dead zones, increasing ocean acidity, and causing thermal stress^1,2^. In order to understand how communities will respond to climate-related stressors, we look to potential deep-time analogues and the community shifts recorded by fossils. The Eocene witnessed two separate long-term warming trends of ~6 °C culminating in the late Ypresian and Bartonian, known as the Early Eocene Climatic Optimum (EECO) and the Middle Eocene Climatic Optimum (MECO), respectively (Fig. S1). Superimposed on these long-term trends are many short-lived intervals of increased carbon injection into the atmosphere and increased sea surface temperatures, known as hyperthermals, and of these the Paleocene-Eocene Thermal Maximum (PETM) and the Eocene Thermal Maximum 2 have the highest magnitude and pace^3^ (Fig. S1). It has been argued that these hyperthermal events represent the best analogues for projected climatic change, as they were caused by rapid increases in *p*CO_2_ and involved various environmental consequences, such as ocean acidification and intensification of the hydrological cycle^2,4^. In addition, the peak of the MECO saw rapid warming^5^ and could be considered another hyperthermal. Similar hyperthermal events of smaller magnitude have also been recorded from the Paleocene, e.g., the latest Danian Event^6^.

The PETM was the most rapid warming event of the early Cenozoic and had the largest ecological impact on marine ecosystems of any hyperthermal during that time^7^. In shallow-water carbonates there was a substantial decline in reef volume^8^ that manifested as a shift from coral-algae reefs to large foraminiferal carbonate ramps^9^; in the deep-sea, there was a major extinction of deep-sea benthic foraminifera^10^, dwarfing of both benthic foraminifera and ostracods^11^; a rapid diversification of pteropods^12^, and poleward shifts in the distribution of planktic foraminifera, dinoflagellates, and radiolarians^13^. Supposed causes of PETM ecological changes include ocean deoxygenation, rising temperatures, shoaling of the calcium carbonate compensation depth, and variations in food supply^7,13^. Nevertheless, the response of non-reefal shallow marine ecosystems to the PETM remains unclear, and there are few studies that quantitatively investigate changes in the composition of macrobenthic assemblages. Along the United States (US) Atlantic Coastal Plain (South Carolina to New Jersey), the PETM interval contains few or no adult molluscs, potentially due to low oxygen conditions and/or ocean acidification^14,15^. Conversely, the US Gulf Coastal Plain (Texas to Georgia) shows no evidence that the PETM resulted in a diversity decline or body size decline in molluscan communities^16,17^. Furthermore, Ivany *et al*.^16^ do not report a faunal turnover at the family-level, but their data do suggest changes in the dominant genera and species.

To improve our understanding of the impact of the early Cenozoic hyperthermals on shallow marine benthic communities, we quantitatively investigated changes in their diversity and composition along the Gulf Coastal Plain. This study tests the hypothesis that early Cenozoic hyperthermal events were associated with significant, long-term changes in community diversity and composition. We compiled a dataset of species abundance and richness from the Late Cretaceous through Eocene (Maastrichtian-Priabonian) interval and quantitatively assessed the molluscan communities for changes in (i) taxon richness, (ii) taxonomic composition, and (iii) functional composition. Although the faunal record does not allow for the assessment of the short-term (up to millennial-scale) responses of molluscs to the early Cenozoic hyperthermals, our comprehensive analysis shows that the early Cenozoic hyperthermals did not significantly impact the evolutionary history of benthic molluscan communities.

## Results

### Diversity changes

**Figure 1:**
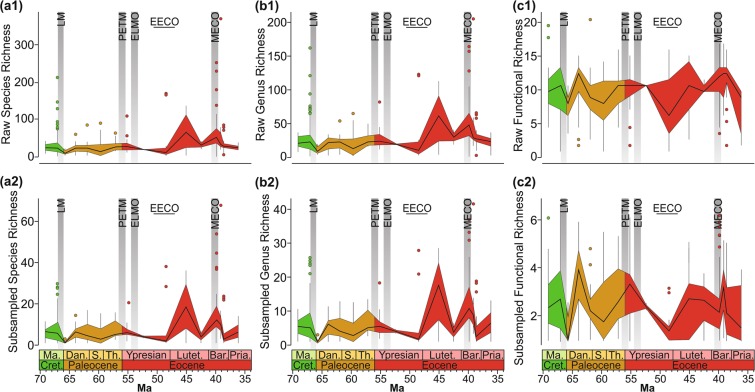
Changes in the diversity of molluscan assemblages during the Late Cretaceous-Eocene interval along the Gulf Coastal Plain. Raw species richness (a1), generic richness (b1), and functional richness (c1) of faunal samples. Subsampled species-level richness (a2), genus-level richness (b2), and functional richness (c2) using the SQS method^17^. Solid black line is the median, the top and bottom of the shaded area corresponds to the first and third quartiles, and the vertical lines represent the lowest and highest datum within 1.5 times of the interquartile range. Points outside of these lines are outliers. The Late Cretaceous and early Cenozoic hyperthermal events discussed in the text are highlighted by vertical grey bars: LM = Latest Maastrichtian, PETM = Paleocene/Eocene Thermal Maximum, ELMO = Eocene Thermal Maximum 2, MECO = Middle Eocene Climatic Optimum. The climax of the early Eocene long-term warming trend is shown by a horizontal bar, i.e., the Early Eocene Climatic Optimum (EECO). Abbreviations: Ma. = Maastrichtian, Cret. = Cretaceous, Dan. = Danian, S. = Selandian, Th. = Thanetian, Lutet. = Lutetian, Bar. = Bartonian, Pria. = Priabonian. Note: samples with <50 specimens were excluded from the analysis. The number of samples in each time interval is shown in Table S1.

**Figure 2:**
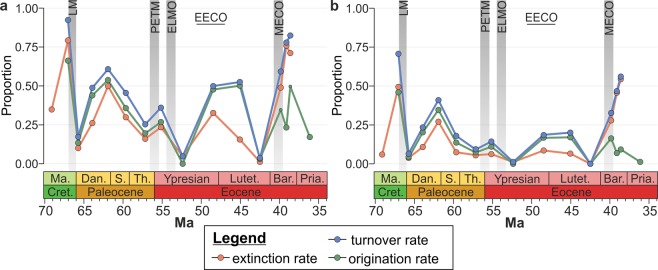
Turnover, extinction, and origination rates during the Late Cretaceous-Eocene interval along the Gulf Coastal Plain. (**a**) Species-level. (**b**) Genus-level. Turnover and origination rates were removed for the first time bin and turnover and extinction rates from the last time bin due to the influence of edge effects. See Fig. 1 for abbreviations.

Analyses of the raw and Shareholder Quorum Sampling (SQS) data yield similar trends in the species, generic, and functional richness of benthic assemblages respectively (Fig. 1). Our analyses provide evidence for two substantial changes in generic richness: a significant (Kruskall-Wallis test (KW): *p* < 0.01), temporary drop at the Cretaceous/Paleogene (K/Pg) boundary (richness quickly recovered by the P1b biozone, ~700 kyr later) and high values of richness in the middle Eocene (Lutetian-Bartonian). The only early Cenozoic hyperthermal with enough samples for statistical inference is the PETM, which does not record a significant change in taxonomic diversity. The late Ypresian samples, which coincide with the Early Eocene Climatic Optimum (EECO), are less diverse than those of other time bins; however, some outliers have a greater richness than other time bins (Fig. 1a,b). The turnover rates show that the decline of genus richness at the K/Pg boundary is associated with a high generic extinction rate, whereas the late Ypresian records a relatively low turnover rate suggesting that the decrease in sample diversity is unlikely to reflect a biotic crisis (Fig. 2). Other than the K/Pg boundary, high turnover rates associated with both elevated extinction and origination of species and genera occur in the late Danian, coincident with the hypothesised late Danian hyperthermal.

Thirty-one different benthic modes of life were recognised in this study, with no more than 21 functional groups in any one sample and no more than six modes of life following SQS (Fig. 1c). In general, the different time bins fall within categories of high, medium, and low functional richness. The intervals that show significant declines in functional diversity belong to the early Danian, late Ypresian, and Priabonian samples (KW: *p* < 0.01) (Fig. 1c). The low functional richness in the early Danian represents a consequence of the end-Cretaceous mass extinction and has been recognised in other datasets^18^. In contrast, low functional richness in the late Ypresian, coincident with the EECO, has not been documented before.

### Compositional shifts

**Figure 3:**
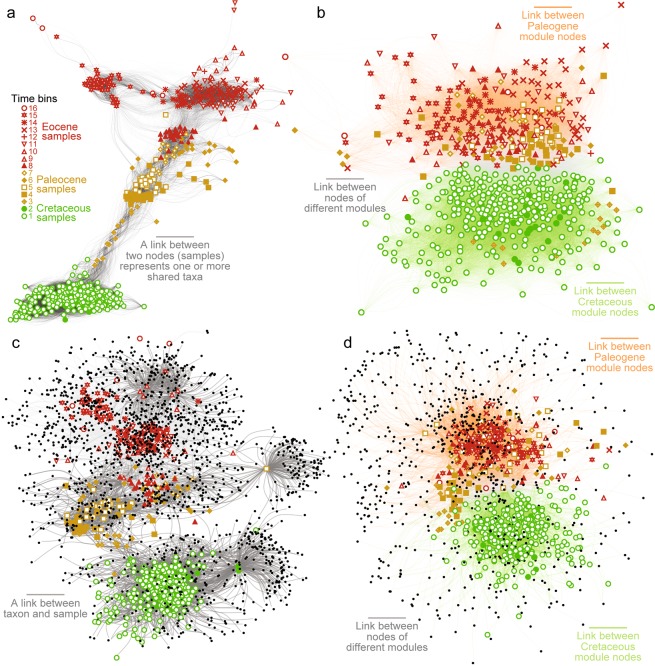
Network analysis of early Cenozoic molluscan communities from the Gulf Coastal Plain. (**a,b**) Unipartite network graphs showing changes in the composition of molluscan assemblages. Two samples are connected if they share one or more taxa, and each connection weight equals the Bray-Curtis similarity of the samples calculated from taxon data. (**a**) Bray-Curtis similarity calculated from species data (modularity, 0.399758). (**b**) Bray-Curtis similarity calculated from genus data (modularity, 0.2434448). In (**b**), modules found with the walktrap community detection algorithm (step length equal to 5) are illustrated with green and orange connections. (**c**,**d**) Bipartite network graphs showing changes in the presence and absence of mollusc taxa among samples. Taxa are connected to their samples, and vice versa, with non-weighted links. The modules found with COPRA method are illustrated with green and orange connections. Black nodes in (**c**) are species (modularity of sample projection, 0.766; modularity of species projection, 0.795), and in (**d**) are genera (modularity of sample projection, 0.417; modularity of species projection, 0.551).

Network analyses of fossil data illustrate compositional differences among samples of different ages, and specifically, how taxa are distributed among the samples^19^ (Fig. 3). We structured the data into unipartite and bipartite networks, each consisting of nodes and links. In the unipartite networks (Fig. 3a,b), each node is a sample; a link indicates that two samples share one or more taxa; and the links are weighted equal to the Bray-Curtis similarity scores of the connected samples^20^. The bipartite networks (Fig. 3c,d), in contrast, contain both sample and taxon nodes where samples are connected by non-weighted links to their taxa, and vice versa, but no two samples (or two taxa) are directly connected to each other^19^. We partitioned these networks into modules (clusters of nodes) using ‘community detection algorithms’^19–22^, which seek to identify the most densely connected nodes separated by the regions with fewest links. The unipartite network was partitioned into non-overlapping modules using weighted random walks, so the results reflect relative abundances of taxa. Conversely, the bipartite network was partitioned into overlapping modules using a non-weighted modularity-based algorithm focused on the presence/absence of taxa^20^. The graphs of these networks illustrate that, although the Cretaceous and Paleogene samples differ with regard to both species and genera, the various time bins of the Paleogene are, by and large, only distinguishable at the species-level. Overall, the algorithms identified numerous clusters at the species level—20 in the unipartite network (Fig. 3a) and 12 in the corresponding bipartite network (Fig. 3c)—but only two modules in the genus-level networks (Fig. 3b,d). These results show that the species-level networks consist of distinct clusters of various Cretaceous, Paleocene, and Eocene ages; the boundaries between these modules correspond to changes in communities around the K/Pg boundary, early Danian, Ypresian, and the late Bartonian. Conversely, each genus-level network has two modules: one composed of Cretaceous and early Danian samples and a second consisting of all remaining Paleocene and Eocene samples (Fig. 3b,d). In terms of taxonomic composition, the amount of overlap between these modules is negligible (Fig. 3d), as the two overlapping modules in the bipartite network share only 15 genera of 797 total (1.9% of the data). The similarity of results for the unipartite and bipartite networks, which are based on relative abundance and presence/absence data, respectively, suggest that the differences between the time intervals primarily reflect changes in the presence/absence of taxa. Several samples of early Danian age are assigned to the first module with the Cretaceous nodes because they evidently consist of genera that survived the K/Pg transition. Beginning at some point in the Danian, the origination/immigration of genera caused significant change in the generic composition of marine communities, as demonstrated by the clustering of all remaining Paleogene samples. Although the Paleocene and Eocene differ to a large degree in terms of species, their samples are indistinguishable in terms of genera. Therefore, network analysis shows that mollusc communities experienced greater compositional change at the species-level than the genus-level throughout the stratigraphic interval of study and that, unlike the end-Cretaceous event, the early Cenozoic hyperthermals did not have a long-term or significant impact on the generic composition of the benthic assemblages.

**Figure 4:**
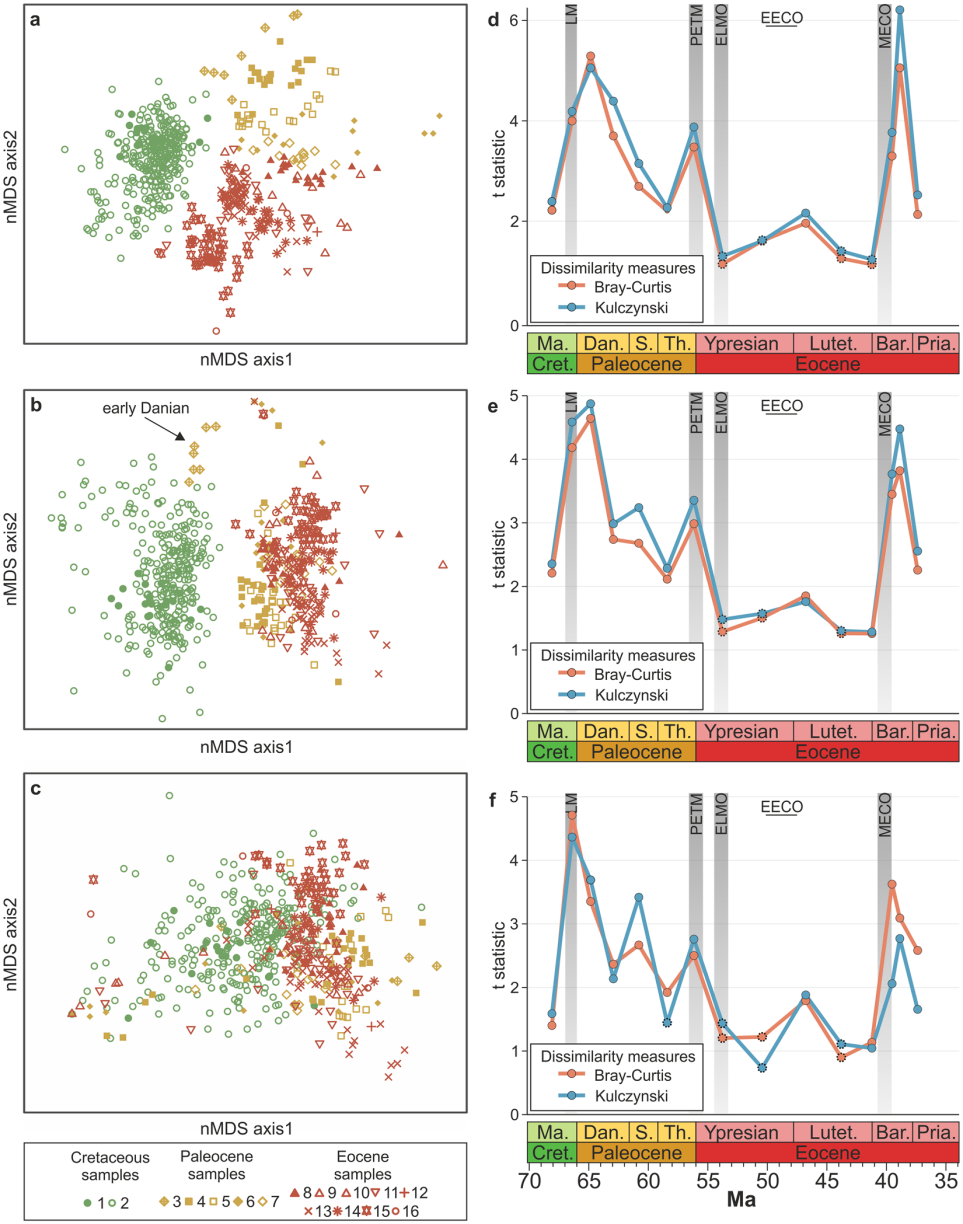
Changes in the composition of molluscan assemblages during the Upper Cretaceous-Eocene interval along the Gulf Coastal Plain. Non-metric multidimensional scaling (nMDS) ordination of molluscan assemblages based on (**a**) species, (**b**), genera, and (**c**) functional compositions. Samples with <50 specimens and individual stress values > 0.3 were excluded. Colours of data points correspond to the different epochs (as in Fig. 1); sample numbering (and symbols) corresponds to the time bins of Fig. S2. T-statistic from the PERMANOVA test using the Bray-Curtis dissimilarity of centroids for relative abundances and Kulczynski dissimilarity of centroids for presence/absence based on (**d**) species, (**e**) genera, and (**f**) functional compositions of sequential time bins: each point represents the dissimilarity between the time bin and the previous time bin. Insignificant *p*-values for the PERMANOVA t-statistic are indicated by dashed circles.

Nonmetric multidimensional scaling (nMDS) ordination based on species composition shows an evolutionary trend with the greatest shifts in species composition at the K/Pg boundary, the Danian, the PETM, and the late Bartonian (Fig. 4a,d). These shifts are also larger than at the genus-level due to the shorter stratigraphic ranges of species. Genus-level compositional changes are reflected in just three clusters: the Maastrichtian, early Danian, and remaining Paleocene-Eocene samples (Fig. 4b). The close resemblance of the Bray-Curtis and Kulczynski dissimilarity measures between centroids suggests that the disparities between intervals are driven by changes in the presence/absence of genera, rather than changes in the relative abundances (Fig. 4e). The change in composition between the Cretaceous and Paleocene occurs in two steps: the first corresponding to the mass extinction event and the second reflecting the evolution/immigration of new genera during the post-extinction recovery (Fig. 4b,e), shown by the high origination rates in the mid-Danian (Fig. 2b). Although clustering close together, a permutational multivariate analysis of variance (PERMANOVA) shows that the Paleocene and Eocene samples are compositionally dissimilar (t = 3.0, *p* < 0.01), suggesting that the PETM resulted in a change in benthic community composition (Fig. 4e). This dissimilarity value is, however, not much higher than the Danian and Selandian values (t = 2.7, p < 0.01; Fig. 4e). In addition, the t-values of the Paleocene and Eocene samples are consistent with high similarities, as demonstrated by nMDS (Fig. 4b).

The nMDS analysis of the functional diversity data did not resolve any clusters of time bins (Fig. 4c). The functional dissimilarity between time intervals does show similar trends, albeit noisier: the highest dissimilarity occurs between the Cretaceous and Danian samples. The similarity between the Kulczynski index compared to the Bray-Curtis index suggests that changes in the presence/absence of modes of life within a sample are causing the differences, rather than the relative abundance of modes of life. The PERMANOVA results show that for the early Danian, late Danian, PETM, and Bartonian, the t-values are highest suggesting functional composition shifts at these times. Unlike changes in taxonomic composition, these shifts do not cause evolutionary changes but temporary changes to a different functional state. Like changes in the taxonomic composition, the early Cenozoic hyperthermals did not cause dissimilarity values greater than in the Paleocene (Fig. 4f), and thus, hyperthermals did not lead to significant restructuring in the functional composition of benthic communities. The PERMANOVA shows that the early Danian samples have a higher dissimilarity (t = 4.7, *p* < 0.01) when compared to the late Maastrichtian and Danian samples, but they still show an overlap. The higher dissimilarity in the early Danian samples is due to their higher abundance of deep-infaunal, facultatively motile, deposit-feeders.

## Discussion

The sequence stratigraphic framework of the Gulf Coastal Plain is characterised by a series of sequence boundaries, condensed units, and variable sedimentation rates that would be expected to overprint the evolutionary history of molluscan communities along the Gulf Coastal Plain^23^. The expected impacts on the fossil record would, therefore, be that first and last appearances are concentrated along sequence boundaries leading to pseudo-extinction and origination events, variations in time-averaging with beds that were deposited during low sedimentation rates having an elevated richness, and rapid facies changes causing punctuated shifts in the assemblage composition^24^. Despite these expected impacts on the fossil record, the only interval in the early Cenozoic with elevated extinction and origination rates is the late Danian, which is not associated with a major hiatus^25^. In contrast, the PETM on the Gulf Coastal Plain does coincide with a sequence boundary, but elevated turnover rates are not recorded (Fig. 2). Likewise, glauconitic lithologies, which are interpreted to have lower sedimentation rates than other lithologies, and thus increased time averaging, are only significantly more diverse than mixed carbonate-clastic rocks but not more diverse than carbonate or clastic rock types (see Supplemental Material). Together, this suggests that at the resolution of our study neither the sedimentology nor the sequence stratigraphic architecture of the Gulf Coastal Plain is overprinting the long-term ecological changes during the early Cenozoic.

Our results indicate that the early Cenozoic hyperthermals did not lead to long-term changes in faunal diversity or composition at the genus-level. In comparison to genera, species generally have shorter stratigraphic ranges, and therefore exhibit higher rates of turnover throughout the stratigraphic interval of study. Consequently, the samples of various ages differ in composition, suggesting that the Cenozoic hyperthermals may have impacted benthic mollusc assemblages at the species-level. Regardless, the species-level turnover rates do not show unusually high values associated with the early Cenozoic hyperthermals. High generic turnover rates are only associated with the late Danian, which could be a consequence of a late Danian hyperthermal. This result stands in stark contrast to observations of the biotic shifts associated with mass extinctions driven by similar environmental changes (e.g., end-Permian, end-Triassic, and the early Toarcian extinction events^26^). Our analyses show that the end-Cretaceous mass extinction and the appearance of new taxa in the Danian caused the only significant shift in the generic composition of benthic communities in the Gulf region during this time (Figs. 2–4). Ivany *et al*.^16^ also showed that the PETM did not lead to lasting changes in body size, species-level diversity, or the life history of dominant benthic clades. These observations suggest that the environmental changes associated with the early Cenozoic hyperthermals, in particular the PETM, were not detrimental to shallow marine molluscan faunas, despite evidence for rapidly rising temperatures, ocean acidification, and deoxygenation^2,7,27^. Our results highlight a major distinction between different ecosystems and environments during the PETM; while the molluscan communities studied here were largely unaffected, others have recorded a notable (50%) extinction of deep-sea benthic foraminifera^28^, a decline in metazoan reef volume and an associated turnover of reef fauna^8,29^, loss of the sedimentary mixed layer^30^, as well as poleward range shifts^13^ and extinctions of nannoplankton^31^. The Gulf Coastal Plain may not record short-term composition changes due to the coarse binning of samples in this study and hiatuses at sequence boundaries, but such potential biases did not diminish the diversity and composition changes associated with the end-Cretaceous mass extinction. Still, if the early Cenozoic hyperthermals led to an increased extinction intensity or the extinction of dominant genera, they would have also resulted in significant composition shifts and elevated turnover rates. Because we did not find significant differences in taxonomic and ecological composition among early Cenozoic samples (Figs. 3 and 4), we conclude that the hyperthermals did not lead to long-term changes in benthic communities. This conclusion does not rule out transient changes in other ecological attributes. An increase in temperature, for example, may reduce body-size, promote parasitism, and/or alter habitat preferences, as demonstrated by work on modern systems^32^ and Mesozoic hyperthermal events^33^. Nonetheless, if any ecological changes did occur during the hyperthermal events in the Gulf Coastal Plain they must have been reversible and short-lived.

**Figure 5:**
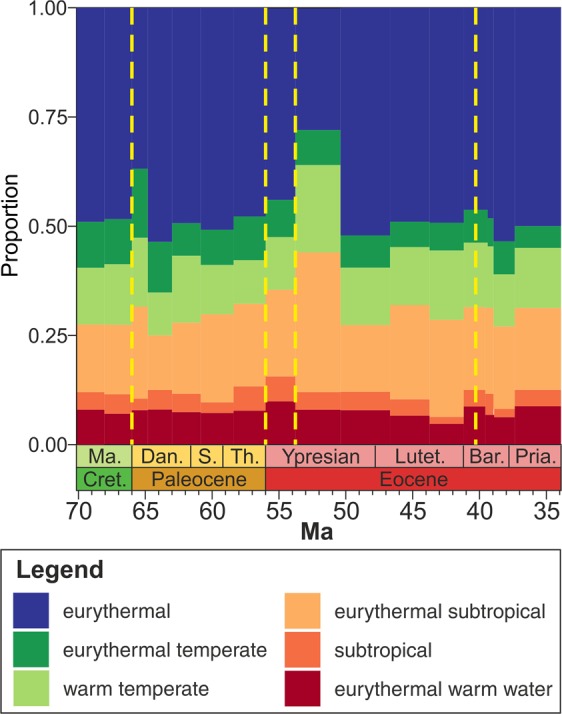
Composition of species from the dataset in each time interval based on their palaeolatitudinal range. Yellow dashed lines represent hyperthermal events discussed in the text, from left to right: late Maastrichtian, PETM, ETM-2, and the MECO. See Supplemental Material and Fig. S5 for the definition of each palaeolatitudinal group. See Fig. 1 for timescale abbreviations.

A plausible explanation for the resilience of benthic marine communities to early Cenozoic hyperthermals could be that communities consisted of genera inferred to have warm-water affinities and thus could tolerate high temperatures^34^. The faunal assemblages of the Gulf Coastal Plain are dominated by gastropods, bivalves, and scaphopods, which have dominated benthic assemblages during hothouse periods since the Mesozoic^35^ and include many taxa with modern representatives that have high-temperature tolerances^36^. Likewise, an investigation of the latitudinal ranges of the species in the Paleocene and Eocene observed in this study shows that ~30-35% of the species have warm water affinities and ~45-50% are eurythermal species that were not recorded north of the warm temperate zone (up to 43 °N) (Fig. 5). These warm water affinities of molluscs may explain why the early Cenozoic hyperthermals caused significant ecological turnover in ecosystems dominated by other faunal groups with lower thermal limits, such as the replacement of corals by large-foraminifera as reef builders^8^. Even though the environmental changes in the Gulf Coastal Plain did not lead to long-term impacts on benthic mollusc communities, other regions that experienced even higher temperatures during the early Cenozoic hyperthermal events may have experienced significant biotic turnover; for example, PETM temperatures of 40 °C in Tanzania caused a temporary exclusion of planktic organisms^37^.

Previous investigations of molluscan extinctions along the Gulf Coastal Plain concluded that changes in temperature drove the disappearance of taxa over time^34,38,39^. These studies show that climatic cooling at the Eocene/Oligocene transition resulted in a regional extinction, including the loss of endemic taxa that were adapted to the high early Cenozoic temperatures^34,38^. The different responses of molluscs on the Gulf Coastal Plain to cooling and warming events highlight the relationships between evolutionary history, ecological structure, and environmental change. The impact of a major environmental state shift on biodiversity, therefore, may depend on the starting state of the system^40^. A rapid warming event in a greenhouse world will have less impact than one in an icehouse world, and vice versa. This interpretation is based on the expectation that species in hothouse worlds would have greater thermal tolerance to high temperature or greater thermal plasticity than species in icehouse worlds. This phenomenon can be seen in modern populations of the gastropods *Chlorostoma funebralis*, which suffer less heat stress and recover quicker from heat stress in hot than in cooler settings^41,42^. Furthermore, given that the species from the Gulf Coastal Plain are mostly warm-temperate (Fig. 5), which tend to have wider thermal niches than tropical species^43^, and provided most live close to their thermal optimum and not at their upper thermal limits (a condition that seems to be more common in tropical species) this offers another explanation for the low impact of hyperthermals. Accordingly, the early Cenozoic hyperthermals do not provide ideal analogues for predicting the consequences of current and projected climate change. Whereas the Paleocene-Eocene events occurred in a hothouse climate, projected climate warming takes place under icehouse conditions that developed following the Oligocene^44^. Consequently, tropical ectotherms are likely more vulnerable to climate change today than they were during the early Cenozoic^45^. In this context, studies of projected climate change will likely underestimate long-term consequences for the biosphere if they rely on the early Cenozoic hyperthermals as frames of reference. Furthermore, projected anthropogenic climate warming – like other events in Earth history such as the end-Permian, and end-Triassic mass extinctions – is not only associated with high magnitude climate warming but also a high rate of temperature change^46^. Even though the magnitude and rates of warming for these ancient episodes of severe environmental perturbation are underestimated^46^, they may be better analogues for assessing the impacts on present-day marine ecosystems^47^.

Apart from heat stress, the development of hypoxic dead zones is a major driver of ecological change in modern shallow marine ecosystems^48^, and is perhaps one of the most significant drivers of extinction during ancient hyperthermal events^49^. The formation and spread of the Gulf of Mexico dead zone in the recent past has caused the complete loss of benthic invertebrates from some regions^50^. In addition, runoff and nutrient-input into the Gulf of Mexico has caused the dead zone to expand and marine diversity to decline^51^. Increases in runoff, nutrient-input, phosphorus regeneration, and dead zone development also affected shallow marine environments during the PETM^14,52^, including those along the Gulf Coastal Plain^53^. Such environmental deterioration possibly also occurred during the other early Cenozoic hyperthermals. Although no lasting impact of any such environmental changes is recorded in the benthic communities studied here, the constraints on temporal resolution in our dataset do not rule out the possibility of expanded, but transient, dead zones during the hyperthermal events. Even if temporary dead zones occurred, benthic communities could rapidly recolonise habitats without significant turnover.

Ocean acidification is another major threat to modern marine communities^2^, and geochemical studies provide support for shoaling of the calcite compensation depth (CCD) and surface ocean acidification during the PETM^2,54,55^. The shoaling of the CCD is thought to have driven the extinction of deep-sea benthic foraminifera during the PETM^28^, and may have even reached unexpectedly shallow depths^15^. Surface ocean acidification may have produced some detrimental effects on benthic fauna (especially for planktotrophic molluscan larvae), but the lack of composition change along the Gulf Coastal Plain, the diversification of pteropods^12,56^, and minimal changes in boron/calcium ratios of planktic foraminifera^12^ suggest that it was not a significant stressor. It is possible that the habitats studied here were too shallow to have been affected by the shoaling CCD during the early Cenozoic and/or that surface ocean acidification may not have developed to lethal levels. The fauna investigated in this study may have also possessed adaptions for coping with ocean acidification. Given that molluscs regulate pH and carbonate chemistry at their calcification sites and possess organic shell coatings^57–59^, the lack of major changes in composition indicates that those genera that dominated the assemblages withstood any effects of adverse environmental deterioration that developed during the early Cenozoic hyperthermals.

## Materials and Methods

We compiled a database of benthic molluscan fossil abundances from the US Gulf Coastal Plain, which were assigned to 16 time intervals that correspond to formations that could be correlated lithostratigraphically (see Figs. 1 and S1, S3). Faunal data come from our own collections and the literature. Our collections comprise 142 samples collected from 129 localities along the Gulf Coastal Plain (Dataset S1). Samples were taken at the bed-level and fossils were collected exhaustively until no further specimens were found. Except for careful cleaning and the removal of matrix, no other preparation was necessary. In total, 111 240 mollusc specimens were identified to the genus-level, and 93 267 were identified to the species-level. Quantitative faunal data from the literature comes from sources that reported original specimen counts^3–13^, increasing the database to 958 samples and 316 767 mollusc specimens (this data is also available from the Paleobiology Database https://paleobiodb.org, and related collection numbers are available in Dataset S1). Taxonomic identifications limited to the family-level were excluded. Samples with <50 specimens were excluded from the analysis, which reduced the dataset to 608 samples. Each genus was assigned to a mode of life within a modified version of the Bambach ecospace model after Sessa *et al*.^60^, which is a combination of tiering, motility, and feeding (see Supplemental Material). The stratigraphic framework for the timing of the early Cenozoic hyperthermals is poorly understood for the Gulf Coastal Plain with only the PETM identified from just below the Tuscahoma/Hatchetigbee Formation boundary^53^. Correlation of the hyperthermals on the Gulf Coastal Plain is, therefore, based on the nannoplankton zonation (Fig. S3).

In our dataset, 32% of the species are informally described (i.e., sp. or spp.), which means that the species variation between samples is likely to be underrepresented in this study. The sample of greatest richness contains 372 species from 212 genera. Recognising that taxonomic richness varies with the number of identified specimens, we normalised the data with respect to sampling effort by applying the Shareholder Quorum Sampling (SQS) method^61^ (SQS; quota = 0.6) using SQS version 3.2^61^ in the statistical programming environment R^62^ to calculate subsampled diversity for each sample. Samples with only a single taxon were excluded when calculating subsampled diversity using the SQS function. For investigating functional diversity, diversity metrics were calculated on the functional groups that were a combination of tiering, motility, and feeding and the abundances of multiple genera within a functional group were tallied for each sample. For each investigated time bin the taxa were characterized as either: confined to interval (FL), only bottom boundary crossed (bL), only top boundary crossed (Ft), or both boundaries crossed (bt) following Foote^63^. From this data the extinction rate ((*N*_*bL*_ + *N*_*FL*_) / total number of taxa), origination rate ((*N*_*Ft*_ + *N*_*FL*_)/total number of taxa), and turnover rate ((*N*_*bL*_ + *N*_*Ft*_ + *N*_*FL*_)/total number of taxa) were calculated.

For multivariate analyses, absolute abundances were converted to relative abundances, since sample sizes were variable throughout the dataset. Some samples were strongly dominated by few genera and so the percentage data were square root transformed to deemphasize the influence of the most abundant genera. Nonmetric multidimensional scaling (nMDS) with the Bray-Curtis similarity matrix was computed to visualise the groupings of samples based on their relative abundances. The stress criterion was used to evaluate goodness-of-fit for the final nMDS ordination^64^. Furthermore, because stress values (the difference between the rank correlation of inter-sample distances and the distances among samples in the ordination space^24^) >0.3 are considered to signify an unsatisfactory representation of the data, those samples with individual stress values > 0.3 were removed from the ordinations. For the species-level analysis, 11 samples were removed prior to the nMDS ordination because they had high stress values (>0.8) and they were distorting the rest of the ordination. A permutational multivariate analysis of variance (PERMANOVA) was carried out to quantify the differences in composition between time bins. These tests were carried out using the Bray-Curtis coefficient for the relative abundance data, and Kulczynski coefficient for the presence/absence data, which are considered the most appropriate coefficients for handling relative and presence/absence data, respectively^64^. The resulting t-values from pairwise tests give an absolute measure of separation between the groups with greater values indicating a greater difference between community structures^65^. The significance of the t-statistic was investigated using *p-*values with a significance level of 0.05. In samples where fewer than 999 permutations could be generated, Monte Carlo *p*-values were employed. PERMANOVA tests were also carried out on a presence/absence version of the dataset using the Kulczynski measure of dissimilarity to investigate if the difference between groups is due to changes in the relative abundances or the presence/absence of taxa.

Unipartite and bipartite networks were partitioned into modules based on the Bray-Curtis similarities of the samples and incidence of taxa among the samples, respectively (see Supplemental Material). The unipartite network was partitioned into non-overlapping (mutually exclusive) modules with the walktrap algorithm^22^ and the statistical significance of the result was confirmed with randomisation testing, which showed that partitioning of randomly generated networks of corresponding size and degree distribution only rarely (<1%) found modules with higher modularity scores^20^; (Supplemental Material). The bipartite network was partitioned into overlapping (non-mutually exclusive) modules with the community overlap propagation algorithm (COPRA^21^). To find the best output, COPRA was run 100,000 times on the bipartite network, and the solution with the highest extended modularity score (calculated from the sample nodes) was recorded^20^; (Supplemental Material).

## Supplementary information

Supplemental Data.

Supplementary Material.
