# Supplementary material for: Resilience of marine invertebrate communities during the early Cenozoic hyperthermals
Source: Sci Rep. 2020 Feb 7;10:2176. doi: 10.1038/s41598-020-58986-5 (PMC7005832; doi:10.1038/s41598-020-58986-5)
Supplement: Supplementary file 2 — Supplementary Material. [file 41598_2020_58986_MOESM2_ESM.docx]

**Supplementary Information for**

**Resilience of marine invertebrate communities during the early Cenozoic hyperthermals**

William J. Foster, Christopher L. Garvie, Anna M. Weiss, A. D. Muscente, Martin Aberhan, John W. Counts, and Rowan C. Martindale

**Corresponding author:** William J. Foster.

Email: [w.j.foster@gmx.co.uk](mailto:w.j.foster@gmx.co.uk)

**This PDF file includes:**

Supplementary text

Tables S1 to S2

Figs. S1 to S5

**Other supplementary materials for this manuscript include the following:**

Database S1

**Supporting Information Text**

**
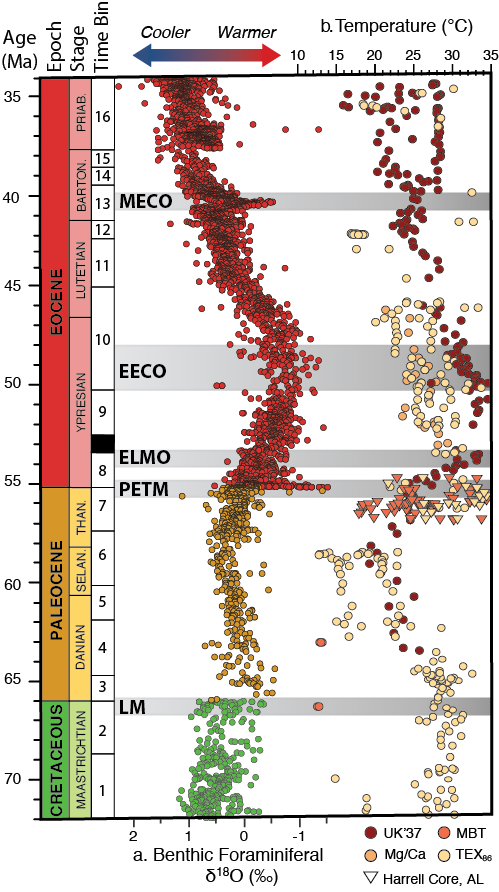
**

**Figure 1 Summary of environmental changes through the study interval with the time bins used in this study**. a) Oxygen isotopes from benthic foraminifera representing changes in deep-ocean temperatures (data from ^1^). b) Sea surface temperatures modified from ^2^. The Late Cretaceous and early Cenozoic hyperthermal events discussed in the text are highlighted by horizontal grey bars: LM = Latest Maastrichtian, PETM = Paleocene/Eocene Thermal Maximum, ELMO = Eocene Thermal Maximum 2, EECO = Early Eocene Climatic Optimum, MECO = Middle Eocene Climatic Optimum. Abbreviations: Selan. = Selandian, Than. = Thanetian, Barton. = Bartonian, Priab. = Priabonian.

**Geological Setting**

The Gulf Coastal Plain is a low-lying region in the southern United States located adjacent to the Gulf of Mexico. Overall, the Gulf of Mexico is an ocean basin that started to form in the Mesozoic during the break-up of Pangaea ^3^. The palaeogeography of the Gulf of Mexico during the Late Cretaceous-Eocene was generally similar to its geography today, in that it was located between North America and the Caribbean Sea, cut-off from the Pacific Ocean by a land mass, and connected to the Atlantic Ocean and Caribbean Sea. The palaeogeography of this region, however, differed from the present configuration, as the Caribbean Sea was connected to the Pacific Ocean, the Florida peninsula had not yet developed, and the presence of the Sabine uplift ^4^. During the early Cenozoic, sea-level was relatively high, so the northern Gulf of Mexico coastline was located further north than today, and southeastern Texas, Louisiana, Mississippi, southern Alabama, and southern Georgia were submerged below sea-level (Fig. S2). Because the Gulf Coastal Plain is a low-lying region, the Cretaceous-Eocene strata containing the studied fossils are mostly exposed around road-cuts, creeks, rivers, and diapiric salt domes. The Cretaceous-Eocene succession consists of the Cretaceous Navarro Group in Texas and the Selma Group in Mississippi, Alabama, and Georgia; Paleocene Midway Group; Paleocene-Eocene Wilcox Group; Eocene Claiborne Group; and Eocene Jackson Group (Fig. S3). Typically, the Navarro and Selma groups are exposed to the northern side of the Midway and Wilcox groups, and the Claiborne Group is exposed south of these groups.


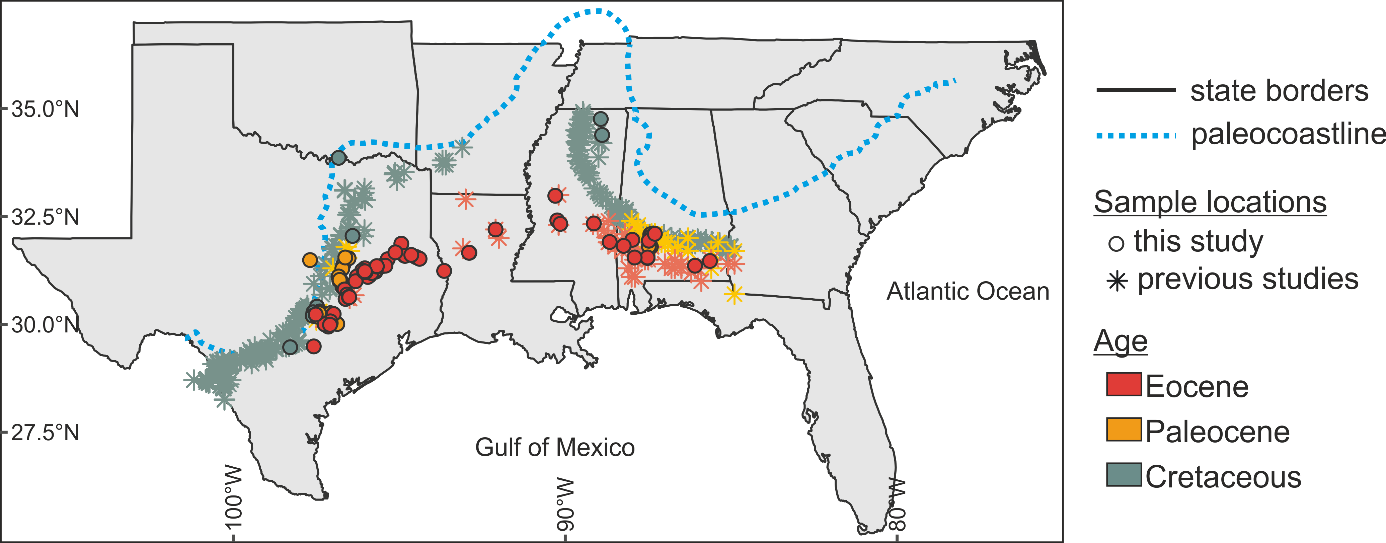


**Figure S2 Map of the 534 sampled locations used in this study.** Circles represent locations from which new data is reported, and the asterisks are locations of previously sampled sites. Green = Cretaceous samples, orange = Paleocene samples, and red = Eocene samples.

**
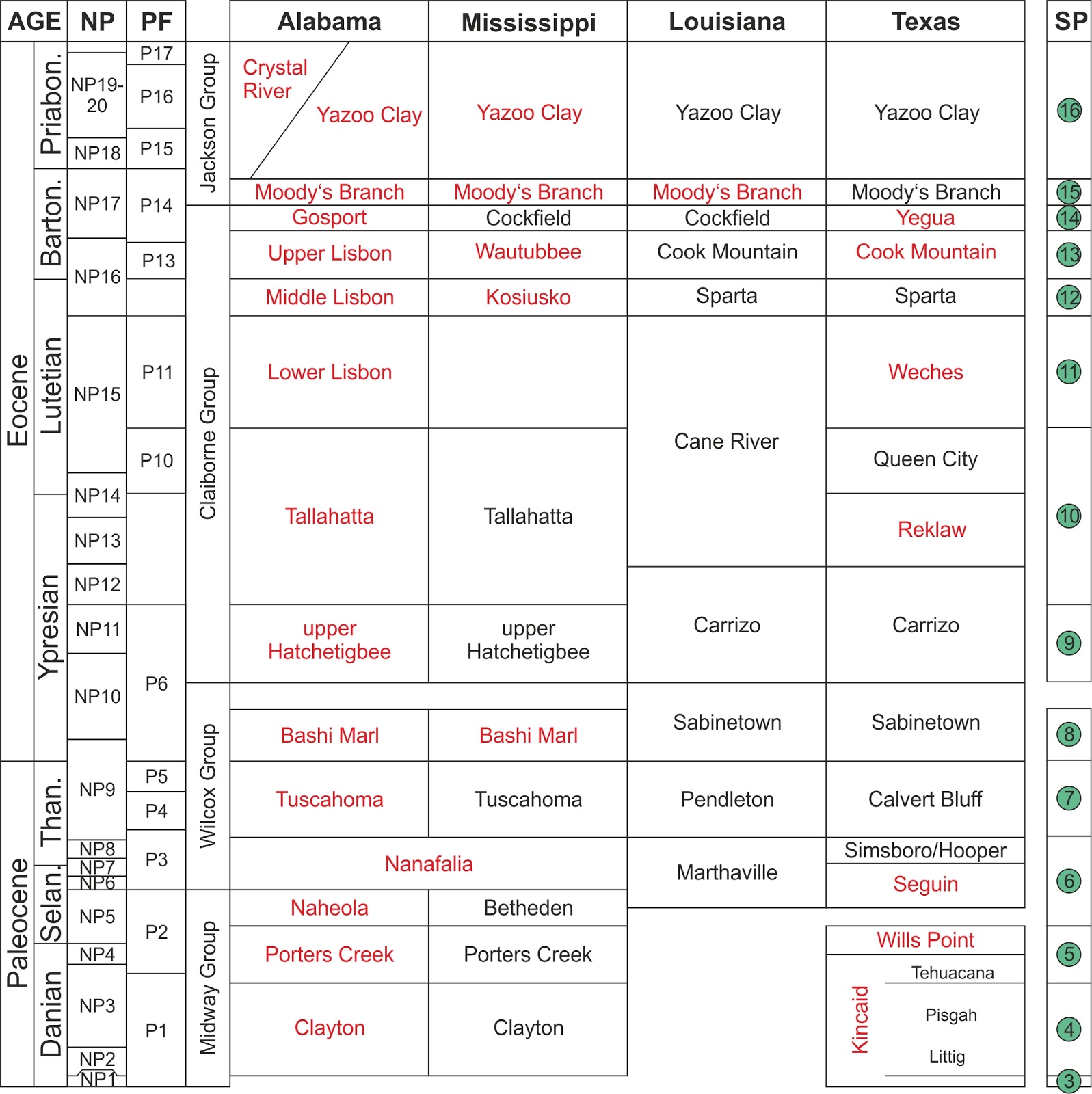
**

**Figure S3 Correlation chart of Paleogene formations in Texas, Louisiana, Mississippi, and Alabama.** NP = Nannoplankton biozone. PF = Planktonic foraminiferal biozone, SP = sample time bins used in this study. Modified after Dockery ^5^, Gaskell ^6^, Mancini and Tew ^7^, Allmon and Ivany ^8^, and Ehret and Ebersole ^9^. Formations that contain samples used in the analysis are highlighted in red. Selan. = Selandian, Than. = Thanetain, Barton. = Bartonian, Priabon. = Priabonian.

**Extended Materials and Methods**

**Database**

Taxonomic assignments for molluscs were updated using MollucaBase ^10^ as it provides an up-to-date authoritative taxonomic revision of molluscs. Generic assignments in open nomenclature, i.e., “cf.”, “[quotation marks]”, “aff.”, “?”, and “informal” were included in the analysis as they only represent 2% of the final dataset, were not shown to significantly alter the results, and represent a more complete picture of the assemblage composition. Cnidarians, bryozoans, annelids, echinoderms, brachiopods, sponges, and arthropods were excluded from the analyses because they are seldom identified to genus-level and are not consistently recorded in the literature.

The ecological assignments of the different taxa: Tiering: **TA_N_** – nektonic; **TA** – sediment-water interface; **TB** – semi-infaunal; **TC** – shallow-infaunal; **TD** – deep-infaunal; **TD_B_** – deep infaunal borers. Motility: **M1** – swimming or fast moving; **M2** – creeping; **M3** – facultatively motile, unattached; **M4** – facultatively motile, attached; **M5** – stationary, unattached; **M6** – stationary, attached. Feeding: **F1** – suspension feeding; **F2** – generalized deposit feeding; **F3** – chemosymbiotic deposit feeding; **F4** – herbivorous grazing; **F5** – browsing predators; **F6** – carnivorous predators. Mode of life assignments were inferred from analogy with living relatives, functional morphology, and previous publications (Dataset S1).

The lithostratigraphical framework across the Gulf Coastal Plain can be divided into groups, formations, and members (Fig. S3). Even though the Paleogene lithological groups can be traced from Texas to Alabama, only the upper Eocene formations occur across the study area. Furthermore, the exposures of upper Cretaceous and lower Cenozoic successions are limited to small outcrops, which makes it impossible to correlate the investigated beds into a continuous succession. We, therefore, correlated the samples from the different formations to the finest resolution, which allows changes across time bins (see Fig. S1) to be analysed.

**Table S1         Number of samples in each time interval used to analyse changes in diversity and faunal composition.** Time bins correspond to figure S1.

| Time Bin | Age | Species-level | Genus-level |
| --- | --- | --- | --- |
| 1 | Maastrichtian | 21 | 21 |
| 2 | Maastrichtian | 293 | 298 |
| 3 | Danian | 13 | 13 |
| 4 | Danian | 25 | 26 |
| 5 | Danian | 24 | 25 |
| 6 | Selandian | 18 | 18 |
| 7 | Thanetian | 14 | 14 |
| 8 | Ypresian | 27 | 27 |
| 9 | Ypresian | 2 | 2 |
| 10 | Lutetian | 16 | 17 |
| 11 | Lutetian | 21 | 21 |
| 12 | Bartonian | 3 | 3 |
| 13 | Bartonian | 54 | 55 |
| 14 | Bartonian | 21 | 21 |
| 15 | Bartonian | 43 | 43 |
| 16 | Priabonian | 5 | 5 |

**Network Analysis**

Networks were visualized in RStudio using functions of the igraph and ggplot2 packages ^11,12^. Nodes of equal size were placed without self-loops according to the Fruchterman-Reingold force-directed algorithm. The networks were analysed for data clustering through application of community detection algorithms ^13,14^. The unipartite networks were partitioned into non-overlapping (mutually exclusive) modules with the walktrap algorithm ^15,16^ using the corresponding function of the igraph package in RStudio ^11^. This algorithm involves performing short random walks along connections between nodes in the network, relying on the principle that such walks will stay within the modules. Random walk lengths are typically set between three and five steps, where each step equals a connection between nodes. Due to its use of random walks, walktrap is a non-deterministic algorithm, and may produce different solutions across multiple runs on a network. Additionally, its results may vary with random walk length. For these reasons, the analysis was repeated many times for the typical step lengths. For each length of three, four, and five steps, the algorithm was applied 10,000 times to the unipartite networks, and the outputs of the various runs were compared. The outputs associated with the best modularity (Q) scores (see below) are reported. To confirm that the outputs did not arise due to chance given the properties of the network ^13^, for each network, we repeated the analyses on 1,000 networks of corresponding size and degree distribution, which were randomly generated with the sample_degseq function of igraph ^11^, and compared the observed and randomly generated outputs with regard to Q. In its simplest form, Q equals the fraction of links that connect nodes of the same modules minus the expected fraction in an equivalent network with a random distribution of connections ^17,18^. Therefore, Q measures the strength of division of a network into modules, and the best results have the highest Q scores ^13,14^. The Q scores of the observed and randomly generated networks in this study were calculated using the modularity function of igraph ^11^. The calculations took into account the weights of the connections ^17^. An observed output is considered statistically significant if its modularity score is higher than 95% of the modularity scores of randomly generated networks of corresponding properties ^13^.

The bipartite networks were partitioned with the community overlap propagation algorithm (COPRA) written in the JAVA programming language using an overlap coefficient equal to 2 ^19^. Unlike the walktrap algorithm, COPRA can be used to partition networks into overlapping modules, which may share any number of nodes ^13^ (the permissible overlap depends on the overlap coefficient, which represents the maximum number of modules per node). It can also be applied to both unipartite and bipartite networks, and in the latter case, each module contains nodes of each of its two sets (e.g., samples and taxa). In general, COPRA relies on an iterative process called label propagation ^20^, which entails randomly assigning a unique label to each node and then repeatedly replacing the label of each node with the most common label among its neighbours until the algorithm achieves its termination criterion (e.g., every node attains the label used by the maximum number of neighbours). Because label propagation proceeds from a random starting state, COPRA is a non-deterministic algorithm, and may produce different solutions across multiple runs on a network. To find the best output, COPRA was run 100,000 times on each bipartite network, and the solutions with the highest extended modularity scores (calculated from the sample nodes) were recorded ^13^.

**Lithology versus Subsampled Richness**

To test if subsampled richness is biased towards a certain rock type, e.g., carbonate, glauconitic, or clastic, we used a Kruskal-Wallis test and pairwise Wilcox tests (using the *benferroni* p.adjust.method) to investigate any significant differences. Box plots of subsampled richness divided into the different rock types and both statistical tests show that only mixed clastic-carbonate rocks record a significant difference in subsampled richness (Fig. S4; Table S2).


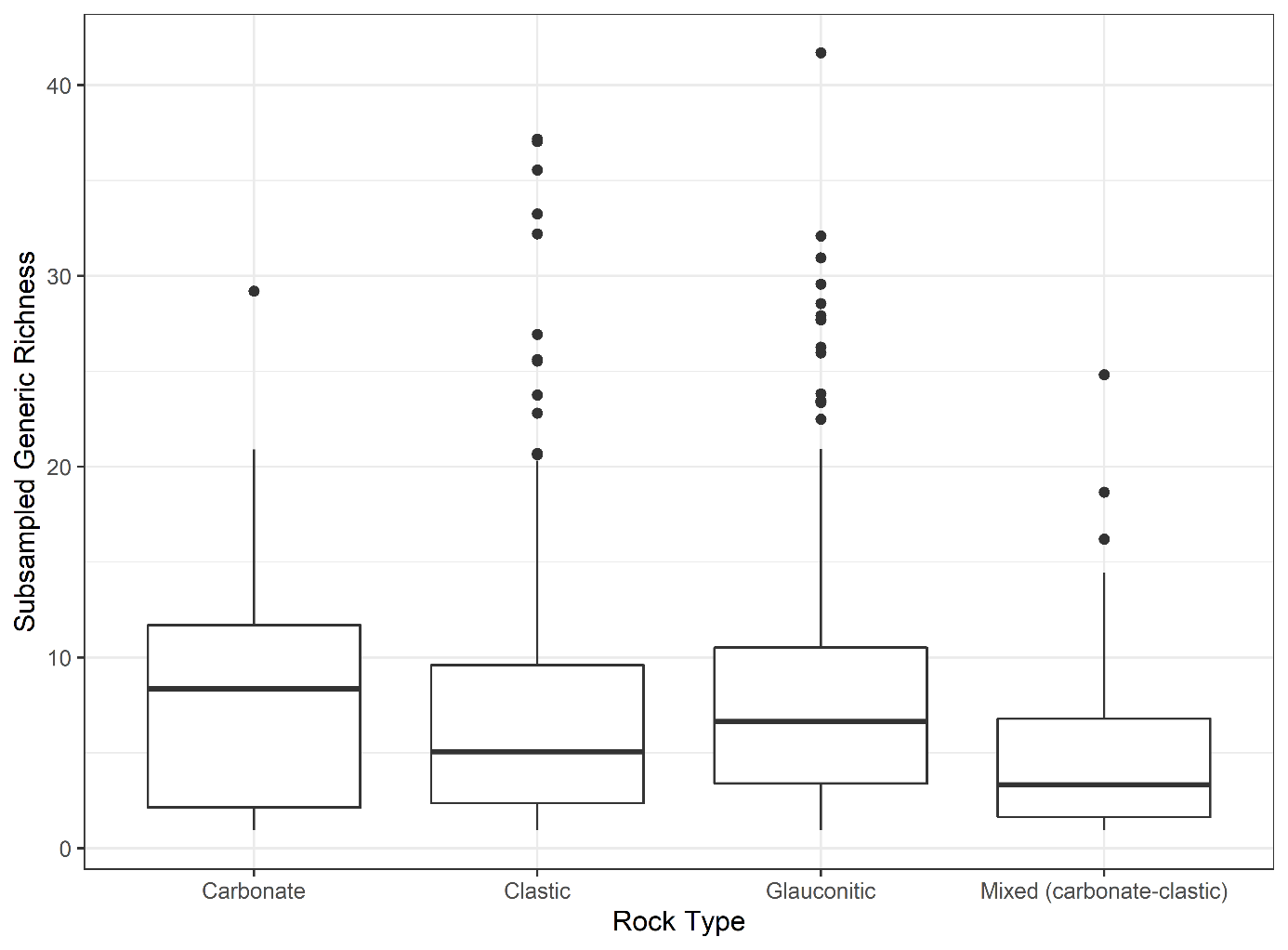


**Figure S4 Box plot of subsampled generic molluscan richness from the Gulf Coastal Plain divided into the four major rock types.**

**Table S2.** P-**values for the pairwise Wilcox test (using the *benferroni* p.adjust.method) of subsampled richness between the different rock types.**

|  | **Carbonate** | **Clastic** | **Glauconitic** |
| --- | --- | --- | --- |
| **Clastic** | 1.00 |  |  |
| **Glauconitic** | 1.00 | 0.07 |  |
| **Mixed (carbonate/clastic)** | 0.02 | < 0.01 | < 0.01 |

**Palaeolatitudinal Species Ranges**

Following Piazza et al. ^21^ it is assumed that the latitudinal range of a species reflects its realised thermal niche. For each species in our dataset from the Gulf Coastal Plain, we downloaded all known occurrences and associated palaeolatitude and palaeolongitude coordinates from the Paleobiology Database (Paleobiodb.org) from the Maastrichtian to the Priabonian. The occurrences were then vetted to be consistent with the taxonomic framework used in this study. Using this data, each species in our dataset was classified as either: subtropical (23° to 35°), eurythermal subtropical (23° to 43°), warm temperate (35° to 43°), eurythermal warm water (0° to 43°), eurythermal temperate (35° to 90°), and eurythermal (0° to 90°) (examples are shown in Fig. S5).


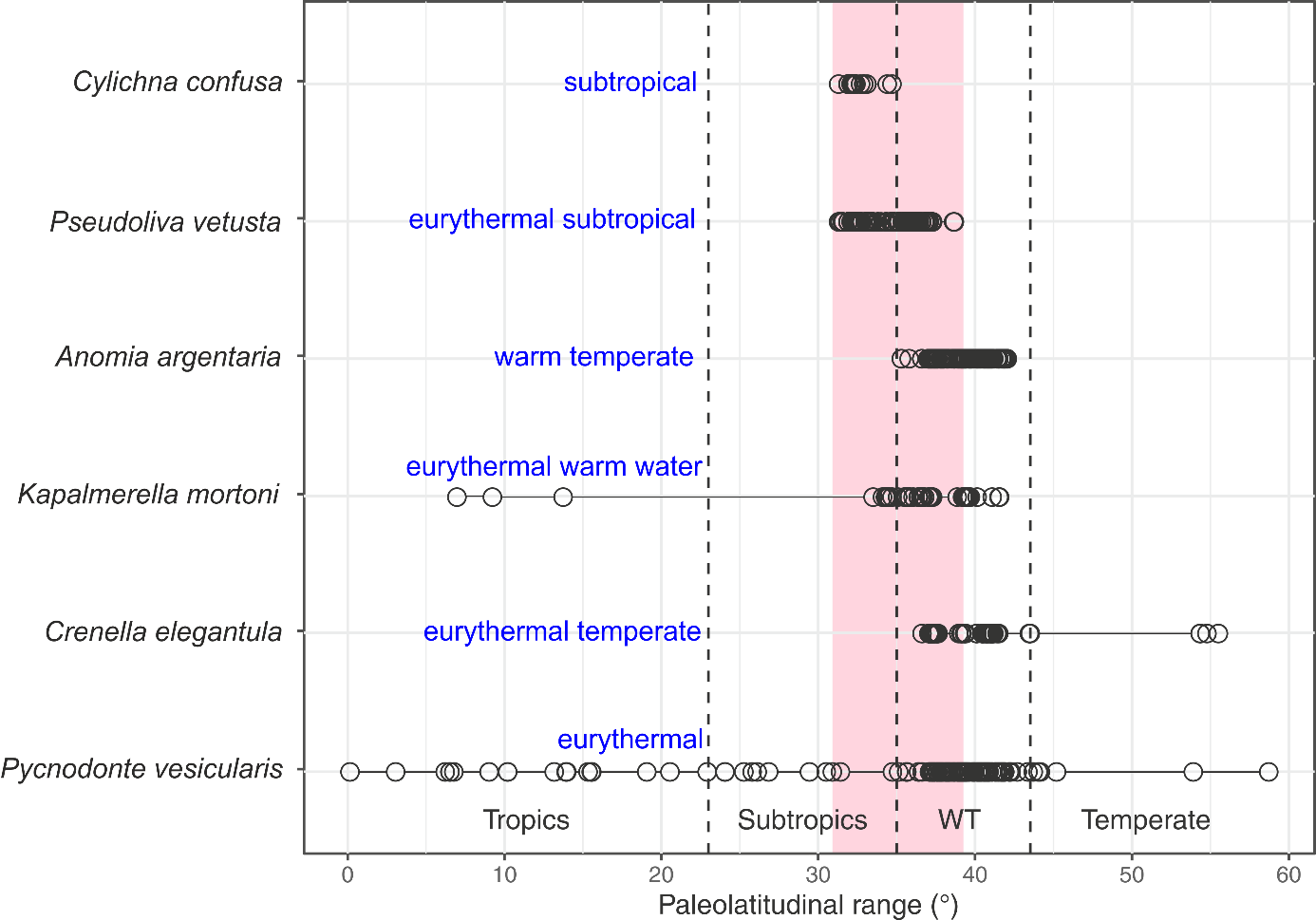


**Figure S5 Examples of the characterization of the thermal tolerances of benthic molluscan species from the Gulf Coastal Plain based on their palaeolatitudinal range. Pink shaded area is equal to the palaeolatitudinal position of the Gulf Coastal Plain during the Early Cenozoic.** Climate zones are separated using dashed black lines with the temperate zone divided at 43° to seperate warmer temperate sea surface temperatures from colder sea surface temperatures. WT = warm temperate. Palaeolatitudinal occurrences come from the paleobiodb.org.

**References**

1 Cramer, B. S., Miller, K. G., Barrett, P. J. & Wright, J. D. Late Cretaceous–Neogene trends in deep ocean temperature and continental ice volume: Reconciling records of benthic foraminiferal geochemistry (δ18O and Mg/Ca) with sea level history. *Journal of Geophysical Research* **116**, 1-23, (2011).

2 Norris, R. D., Turner, S. K., Hull, P. M. & Ridgwell, A. Marine ecosystem responses to Cenozoic global change. *Science* **341**, 492-498 (2013).

3 Salvador, A. *Decade of North American Geology. The Geology of North America. Vol. J. The Gulf of Mexico Basin*. (Geological Society of America, 1991).

4 Blakey, R. C. & Ranney, W. D. *Ancient Landscapes of Western North America: A Geologic History with Paleogeographic Maps (1st edition)*. (Springer Nature, 2018).

5 Dockery, D. T. in *Late Paleocene-early Eocene climatic and biotic events in the marine and terrestrial records* (ed M-P Aubry et al.) 296-322 (Columbia University Press, 1998).

6 Gaskell, B. A. Extinction patterns in Paleogene benthic foraminiferal faunas: relationship to climate and sea level. *PALAIOS* **6**, 2-16 (1991).

7 Mancini, E. A. & Tew, B. H. Relationships of Paleogene stage and planktonic foraminiferal zone boundaries to lithostratigraphic and allostratigraphic contacts in the eastern Gulf Coastal Plain. *The Journal of Foraminiferal Research* **21**, 48-66 (1991).

8 Allmon, W. D. & Ivany, L. C. Testing for causal relationships between environmental and evolutionary change in the marine Paleogene of the US Gulf Coastal Plain: The nature of the problem. *Gulf Coast Association of geological Societies Transactions* **58**, 25-48 (2008).

9 Ehret, D. J. & Ebersole, J. Occurrence of the megatoothed sharks (Lamniformes: Otodontidae) in Alabama, USA. *PeerJ* **2**, e625, (2014).

10 MolluscaBase. *MolluscaBase*, http://www.molluscabase.org/ 2018).

11 Csárdi, G. & Nepusz, T. The igraph software package for complex network research. *InterJournal, Complex Systems* **1695**, 1-9 (2006).

12 Wickham, H. *ggplot2: Elegant Graphics for Data Analysis*. 213 (Springer-Verlag New York, 2009).

13 Muscente, A. D. *et al.* Ediacaran biozones identified with network analysis provide evidence for pulsed extinctions of early complex life. *Nat Commun* **10**, 911, (2019).

14 Muscente, A. D. *et al.* Quantifying ecological impacts of mass extinctions with network analysis of fossil communities. *P.N.A.S* **115**, 5217-5222, (2018).

15 Pons, P. & Latapy, M. in *Computer and Information Sciences - ISCIS 2005.* (eds pInar Yolum, Tunga Güngör, Fikret Gürgen, & Can Özturan) 284-293 (Springer Berlin Heidelberg).

16 Kocsis, A. T., Reddin, C. J. & Kiessling, W. The biogeographical imprint of mass extinctions. *Proc Biol Sci* **285**, (2018).

17 Clauset, A., Newman, M. E. J. & Moore, C. Finding community structure in very large networks. *Physical Review E* **70**, 1–6 (2004).

18 Nicosia, V., Mangioni, G., Carchiolo, V. & Malgeri, M. Extending the definition of modularity to directed graphs with overlapping communities. *Journal of Statistical Mechanics: Theory and Experiment* **2009**, 1–22 (2009).

19 Gregory, S. Finding overlapping communities in networks by label propagation. *New Journal of Physics* **12**, 103018, (2010).

20 Raghavan, U. N., Albert, R. & Kumara, S. Near linear time algorithm to detect community structures in large-scale networks. *Physical Review E* **76**, 1–12 (2007).

21 Piazza, V., Duarte, L. V., Renaudie, J. & Aberhan, M. Reductions in body size of benthic macroinvertebrates as a precursor of the early Toarcian (Early Jurassic) extinction event in the Lusitanian Basin, Portugal. *Paleobiology* **45**, 296-316, (2019).
